# Mechanical Properties and Functional Assessment of PMMA Bone Cements Modified with Glassy Carbon

**DOI:** 10.3390/jfb16070254

**Published:** 2025-07-09

**Authors:** Robert Karpiński, Jakub Szabelski

**Affiliations:** 1 Department of Machine Design and Mechatronics, Faculty of Mechanical Engineering, Lublin University of Technology, Nadbystrzycka 36, 20-618 Lublin, Poland; 2 Institute of Medical Sciences, The John Paul II Catholic University of Lublin, Konstantynów 1H, 20-708 Lublin, Poland; 3 1st Department of Psychiatry, Psychotherapy and Early Intervention, Medical University of Lublin, Gluska 1, 20-439 Lublin, Poland; 4 Department of Computerization and Production Robotization, Faculty of Mechanical Engineering, Lublin University of Technology, Nadbystrzycka 36, 20-618 Lublin, Poland

**Keywords:** composites, bone cement, PMMA, glassy carbon, mechanical properties, compressive strength, bio-integration, functional biomaterials

## Abstract

Poly(methyl methacrylate) (PMMA)-based bone cements are widely used in orthopaedic surgery, yet their inherent brittleness, lack of bioactivity, and exothermic polymerization remain critical limitations. Recent strategies have focused on modifying PMMA with functional additives to improve not only mechanical performance but also thermal behaviour and biological interactions. This study investigates the mechanical properties of two commercial PMMA cements—Palamed^®^ (antibiotic-free) and Refobacin Plus G (gentamicin-loaded)—reinforced with glassy carbon (GC) particles of two different grain sizes (0.4–1.2 µm and 20–50 µm) and various concentrations. The results demonstrate that coarse GC particles (20–50 µm) significantly reduced compressive strength, particularly in the antibiotic-loaded cement. In contrast, the incorporation of fine GC particles (0.4–1.2 µm) did not markedly impair mechanical performance in Palamed^®^, suggesting better compatibility with the PMMA matrix. In addition to mechanical enhancement, the structural and chemical stability of glassy carbon may contribute to improved biological response and reduced polymerization heat. These findings highlight the potential of glassy carbon as a functional additive for designing PMMA-based biomaterials that combine improved mechanical properties with favourable characteristics for long-term implant integration.

## 1. Introduction

Polymethylmethacrylate (PMMA)-based bone cements are widely used in orthopaedics as a material for fixing endoprostheses and filling bone defects. Since their first applications in the 1960s, they have become an essential component of procedures such as joint prosthetics and percutaneous stabilisation of vertebral fractures (vertebroplasty, kyphoplasty, etc.) [1,2,3,4,5,6]. The popularity of PMMA cements is mainly due to their favourable functional characteristics: once mixed, the two-component cementitious mass transforms into an in situ solid state within several minutes, providing primary implant stabilisation in bone only a short time after implantation [7,8,9,10,11]. Although these materials have good working properties and adequate initial strength, they also exhibit significant limitations in a number of respects. First and foremost, they are biologically inert—PMMA does not chemically bond to the surrounding bone tissue, which means that the implant lacks bioactivity and osteointegration. In addition, these cements are characterised by relatively low fatigue strength and brittleness, which in the long term can lead to cement fractures and implant loosening, resulting in the need for revision surgery, which is directly associated with inconvenience and risk for the patient, in addition to generating high costs [12,13]. The modern approach to the design of biomaterials require not only improving their mechanical performance but also considering their ability to integrate with host tissue, heat conductivity, chemical stability, and minimising inflammatory reactions, which fits into the concept of functional biomaterials with optimised structure and bio-interaction. PMMA is added to gentamicin to mitigate postoperative infections through sustained local release. While this approach is clinically important, it may interfere with other cement components, including carbon-based additives, thus impacting polymerisation kinetics, mechanical properties, and long-term stability. In this context, the functional performance of PMMA cements must be considered holistically, as both mechanical reinforcement and antimicrobial function contribute to their classification as functional biomaterials [14,15,16].

Another challenge is the highly exothermic polymerisation reaction: considerable heat is released during the setting of the PMMA cement, which can cause tissue damage (thermal necrosis) in the area surrounding the implantation site [12]. In addition, the hardened cement’s stiffness is not fully matched to the elasticity and mechanical parameters of the bone, resulting in localised stress concentration at the implant–cement–bone interface [12,17].

As a consequence of these limitations, an intensive search is being carried out for modifications to the composition of bone cements that could improve both their mechanical properties, increase bioactivity, and reduce the negative thermal effects that can threaten the surrounding tissues of the implant [18].

A variety of additives and admixtures to PMMA cements have been investigated to date, including bioactive ceramics (e.g., hydroxyapatite particles [19,20,21], tricalcium phosphate (TCP) [22,23], or bioactive glass [24,25]) to provide osteoconductive properties; carbon, glass, or polymer fibres to increase fracture toughness; as well as nanoparticles and nanofibers to enhance and modify other characteristics of the cement and ultimately improve its final performance in both the short and long term [26,27,28].

In the past few years, modifications using nanostructured forms of carbon, such as carbon nanotubes (CNTs) and graphene and its derivatives, have been of particular interest. These materials exhibit a unique combination of high mechanical strength, high specific surface area, and high thermal and electrical conductivity, making them attractive candidates for PMMA reinforcement additives [29,30].

Among the nanostructured carbon additives, carbon nanotubes (CNTs) are currently one of the most widely studied admixtures for the reinforcement of PMMA bone cements. Due to their exceptional mechanical strength and high elastic modulus, even their minor addition (of the order of 0.1–0.5 wt.%) can significantly increase the flexural, fracture, and fatigue resistance of the cement, mainly due to the mechanism of bridging microcracks and effectively transferring loads to the PMMA matrix [31,32,33]. In addition, nanotubes exhibit very high thermal conductivity, which allows for the dissipation of heat generated during exothermic polymerisation, potentially reducing the risk of thermal necrosis in tissues [33]. However, a key challenge remains their uniform dispersion—pure CNTs tend to agglomerate, so it is common to use chemical functionalisation (e.g., with -COOH groups), which improves their compatibility with the matrix and increases the efficiency of mechanical reinforcement [34,35,36]. Despite the positive in vitro results, further research is needed on the biocompatibility and safety of nanotubes under in vivo conditions.

Graphene and its derivatives, in particular graphene oxide (GO), constitute another group of high-performance carbon admixtures being investigated for the reinforcement of PMMA cements [37]. Due to its high specific surface area and the presence of functional groups, GO can be easily dispersed in methacrylate resin, resulting in improved flexural, compressive, and fracture strengths even at low concentrations (0.1–0.5 wt.%) [29]. Graphene additives also show the ability to reduce the exothermic setting of cement due to their good thermal conductivity and radical absorption properties, which slows down polymerisation and reduces the maximum temperature [29]. In addition, some studies suggest that GO can promote osteoblastic cell adhesion and proliferation, especially in the presence of bioactive additives, making it an interesting candidate for creating cements with potentially higher osteointegration [38]. However, it should be noted that, as with CNTs, issues of optimising the content and controlling the interaction with the PMMA matrix remain important.

Among various carbon-based additives, glassy carbon has emerged as a particularly promising yet underexplored material. Due to its structural integrity, biocompatibility, and ability to dissipate heat, it may simultaneously address the major limitations of PMMA bone cements—mechanical fragility, lack of bio-integration, and thermal risks during polymerization—positioning it as a candidate for next-generation functional bone cement formulations. Glassy carbon is an interesting alternative to the traditional carbon admixtures previously used in PMMA bone cements due to its unique physicochemical properties. It exhibits high biocompatibility, chemical resistance, and porous structure, which may promote integration into bone tissue [39,40,41]. Studies have shown that the addition of glassy carbon to PMMA cement affects its mechanical and surface properties. For example, Choryłek and Postawa [42] observed that modification of PMMA cement with glassy carbon leads to an increase in the hardness of the material by approximately 50%, which may be beneficial in the context of handling high momentary loads, such as patient falls. However, it has also been noted that the addition of glassy carbon can cause increased brittleness of the material and the formation of microcracks under compressive forces, which are undesirable in medical applications [43].

The review of the existing literature clearly indicates that carbon modifications of PMMA bone cements represent a promising research direction but require further in-depth studies. Carbon nanotubes and graphene have already partially proven their reinforcing capabilities and beneficial effects on exothermic control, but their practical application is limited by mass-scale homogenisation problems and incompletely resolved biosafety issues (potential cytotoxicity of free nanoparticles). Currently, the most intriguing yet least explored approach appears to be the use of glassy carbon, a material with well-established biocompatibility that could solve some of the problems associated with nanoparticles. So far, there is very little research work with glassy carbon; in particular, there is a lack of data on the long-term effects of its presence in cement, its effect on fatigue, its residual monomer release, and its behaviour under biological conditions.

To conclude, the motivation for our experimental research on glassy carbon PMMA cements is the significant knowledge gaps regarding this concept and its potential advantages related to biocompatibility, good thermal conductivity, and stability of this material. The results of literature studies to date encourage researchers to find out whether, through an appropriate choice of parameters such as particle size, weight proportion, and possible combination with other solid additives such as ceramics or bone fragments, it is possible to obtain a cement with better mechanical and thermal properties without losing biological safety [29,36,38]. This paper attempts to answer these questions. The following chapters present the experimental design and results of our own research, aiming to assess the suitability of glassy carbon as an admixture in acrylate bone cements. This study also attempts to assess the potential of this material as a functional component of a biomaterial that, with appropriately selected structural and chemical properties, can promote integration into the surrounding tissue and improve clinical safety by reducing the effects of the exothermic polymerisation reaction. Therefore, the aim of this study is to evaluate the effect of glassy carbon (GC) particle size and concentration on the mechanical performance of PMMA-based bone cements and to assess the potential of GC as a functional additive that may enhance structural integrity and support biological integration.

## 2. Materials and Methods

### 2.1. Materials

In this study, we evaluated select basic mechanical properties of two commercially available poly(methyl methacrylate) (PMMA)-based bone cements—Palamed^®^ (Heraeus Medical GmbH, Wehrheim, Germany) and Refobacin Plus G (produced by AAP Biomaterials GmbH & Co. KG, Dieburg, Germany; distributed by Zimmer Biomet, Warsaw, IN, USA)—modified with glassy carbon (GC) particles at different concentrations and two granulation ranges, respectively: 20–50 µm and 0.4–1.2 µm (Alfa Aesar, Thermo Fisher Scientific, Karlsruhe, Germany) [44].

Palamed^®^ is a bone cement for endoprosthesis fixation, characterised by high fatigue strength and good processability. It is a low-viscosity product, which facilitates its application and improves penetration deep into the bone beads allowing micro-embedding. The cement does not contain antibiotic additives, making it suitable for studies on the properties of the modified material without interference from bioactive agents.

Refobacin Plus G is a bone cement containing the antibiotic gentamicin, which provides antimicrobial activity in situ. It is a medium-viscosity cement commonly used in orthopaedics for procedures requiring simultaneous implant stabilisation and infection prevention.

**Figure 1:**
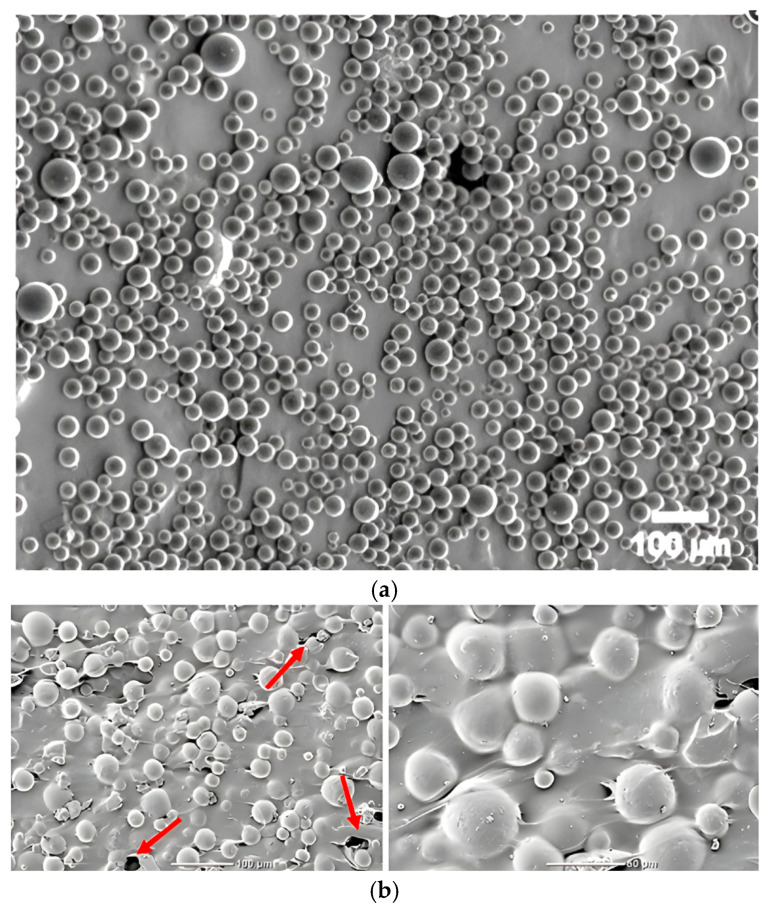
SEM images: (**a**) GC by Structure Probe, Inc. West Chester, PA, USA; (**b**) GC incorporated in PMMA matrix.

Particles of glassy carbon, known for their high biocompatibility, chemical inertness, and excellent mechanical properties such as high stiffness, fracture resistance, and low density, were used as an admixture. This material combines the properties of ceramics and carbon materials, making it an attractive addition to biomaterial composites. Two grain fractions were used in the study: microparticles of 20–50 µm and submicroparticles of 0.4–1.2 µm. The granulation variation was designed to assess the effect of GC particle size on the mechanical properties of the cement, including its compressive strength. Sample SEM images of pure GC and GC incorporated in the PMMA matrix are presented in (Figure 1) [30,45]. Glassy carbon is formed by pyrolysis of organic polymers, carried out at high temperature in an oxygen-free atmosphere. It is characterised by an amorphous, isotropic structure, being a combination of basal and edge planes typical of graphite but arranged in an irregular and disordered manner [46,47]. To ensure precise characterization of the reinforcing phase, the glassy carbon (GC) particles used in this study were obtained from Alfa Aesar (Thermo Fisher Scientific GmbH, Karlsruhe, Germany) and corresponded to two distinct size fractions: coarse (20–50 µm) and fine (0.4–1.2 µm) [44,48]. According to the manufacturer’s specifications, both fractions exhibit a bulk density of approximately 0.47 g/cm^3^, true density of 1.42 g/cm^3^, and high specific surface area (>40 m^2^/g for fine GC). The particles are amorphous and isotropic, with low ash content (<0.01%) and high carbon purity (>99.9%). Their thermal conductivity is rated at 5–6 W/m·K, and they possess excellent oxidation resistance in inert environments [44,48]. SEM micrographs provided by the manufacturer confirm their irregular, granular morphology with sharp-edged contours. These properties are consistent with the expected behaviour of GC in PMMA matrices and support its selection as a candidate for reinforcement in bone cement composites.

The selection of Palamed^®^ and Refobacin Plus G bone cements was intentional and based on their differing characteristics—Palamed^®^ as a representative of antibiotic-free, low-viscosity cements with high fatigue strength and Refobacin Plus G as a clinically relevant, gentamicin-loaded formulation used in infection-prone orthopaedic settings. This comparative approach was designed to assess the effect of GC addition not only across different particle sizes but also in materials with distinct viscosity, bioactivity, and matrix composition. The use of two grain sizes (0.4–1.2 µm and 20–50 µm) and a narrow concentration range (0–5%) was dictated by preliminary studies and literature data indicating this as the effective working range for observing structural modifications without compromising the matrix’s integrity or setting behaviour. A broader scope involving higher concentrations was avoided due to risks of excessive porosity and particle agglomeration, which may induce defects rather than beneficial reinforcement.

### 2.2. Sample Preparation

**Figure 2:**
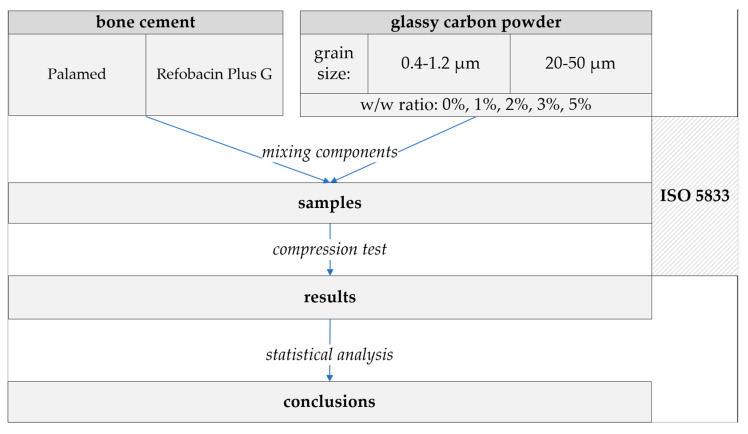
Sample preparation flowchart.

**Figure 3:**
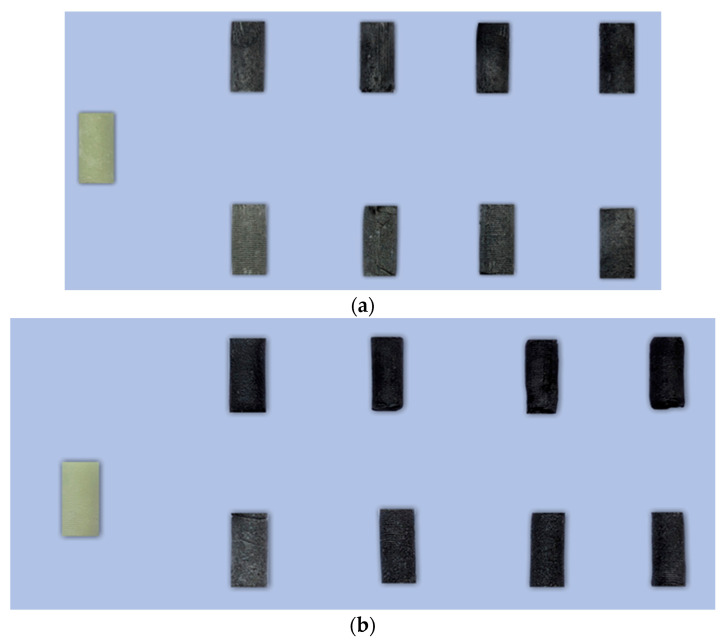
Photograph of representative specimens: (**a**) Refobacin and (**b**) Palamed G; left sample—non-admixed. Top rows: 0.4–1.2 µm GC; bottom rows: 20–50 µm GC. Consecutive columns represent admixture *w*/*w* ratios of 1%, 2%, 3%, and 5%.

Bone cement samples were prepared according to the guidelines of the standard ISO 5833:2002—Implants for surgery—Acrylic resin cements [49]. Both the reference samples (unmodified) and those modified with glassy carbon (GC) particles were produced using a standardised moulding and machining protocol. To ensure repeatability and compliance with ISO 5833:2002 standards, the sample preparation process was carried out in multiple controlled steps. Figure 2 illustrates the schematic workflow of the preparation process, while Figure 3 presents representative images of the prepared specimens with both particle size variants.

After the cement components were thoroughly mixed according to the manufacturer’s recommendations, the appropriate amount of glassy carbon was introduced into the mixture, depending on the planned test batch (0%, 1%, 2%, 3%, and 5% *w*/*w*). The GC particles were dosed at the specified concentration by weight (relative to the weight of the polymer powder) and manually homogenised for 60 s, followed by mechanical mixing for a further 120 s under controlled humidity and temperature conditions. The mixture was placed in cylindrical moulds made of plastic with high dimensional accuracy. The cement was applied to the moulds immediately after mixing, using moderate pressure to minimise the presence of voids and fill the moulding spaces completely. The curing process was carried out at room temperature (23 ± 1 °C), under dry and stationary conditions.

Once the cement had fully cured, the specimens were removed from the moulds and subjected to a careful finishing process. Cylindrical specimens with a diameter of 6.0 ± 0.1 mm were shortened to a length of 12.0 ± 0.1 mm using a precision grinder with an abrasive disc. This operation was aimed at obtaining parallel, flat faces, which is necessary to ensure proper force distribution during mechanical testing, especially in axial compression tests.

The dimensions of each specimen were controlled using a digital calliper with an accuracy of 0.01 mm. The diameter was measured in two perpendicular directions on at least two cross-sections along the specimen, and the obtained values were averaged. For each material combination tested (cement type, fraction, and GC concentration), at least five samples were prepared according to ISO 5833. In practice, the number was usually 8–10 specimens to increase the statistical accuracy of the results and to allow the rejection of any samples that were not of the required quality.

### 2.3. Mechanical Testing

Compressive strength tests were carried out using the MTS Bionix^®^ Tabletop Test System (MTS Systems Corporation, Eden Prairie, MN, USA), a servohydraulic test system that allows the application and precise measurement of compressive force. Pre-prepared cylindrical specimens were placed directly between the working plates of the machine without the use of spacers, allowing the axial load to be transferred evenly.

During the axial compression test, the loading force values were recorded as a function of the crosshead displacement, allowing subsequent development of force–strain curves. The compression speed was kept constant at 20 mm/min [50].

All tests were carried out at room temperature (23 ± 1 °C), in accordance with the requirements of the ISO 5833 standard. The experiment was continued until mechanical failure of the specimen (fracture of the cylindrical specimen) or until one of the strength limits was reached: upper yield strength or force at a displacement corresponding to 2% permanent deformation (offset) [51]. The lowest of these values was taken as the final value. The maximum force recorded was related to the original cross-sectional area of the specimen (calculated from the diameter) to obtain the compressive strength, expressed in megapascals (MPa).

In addition, the slope of the initial (line) section of the curve was determined from the force–deformation time diagrams, which were used to calculate Young’s modulus, i.e., a parameter describing the material’s stiffness. A high value of Young’s modulus means that the material is less susceptible to deformation under compressive forces, indicating greater stiffness and less elasticity of the analysed composite.

### 2.4. Statistical Analysis

All results obtained were subjected to statistical analysis using TIBCO Statistica 13.3 software (TIBCO Software Inc., Palo Alto, CA, USA) to demonstrate statistically significant differences between the values of the tested mechanical parameters of the cements, depending on the concentration and granulation of the glassy carbon (GC) admixture used. The analysis included multi-group comparisons, taking into account the differences between each series of samples.

In order to interpret the data accurately, post hoc tests available in the Statistica package were used, such as Duncan’s multiple range test (MRT), Scheffé’s method, Tukey’s test and Dunn’s test. The choice of a particular comparison method depended on the characteristics of the data, including the size of the groups, the distribution of the variables, and meeting the assumption of homogeneity of variance. These tests differ in their degree of conservatism and resistance to violations of statistical assumptions. Some of them (e.g., the Tukey test) allow the comparison of groups with different group sizes and heterogeneous variances, which makes them particularly useful in the analysis of mixed experimental data.

For series where the number of samples differed between comparison groups, the Tukey multiple comparisons test was applied. Prior to its use, a standard verification of the meeting the basic statistical prerequisites, i.e., normality of distribution (using the Shapiro–Wilk test) and equality of variance (based on the Levene’s or Bartlett’s test), was carried out in accordance with the current methodological guidelines [52].

In all analyses, a standard significance level of α = 0.05 was adopted, considering differences between groups to be statistically significant if the *p*-value was less than the adopted α-level.

## 3. Results and Discussion

In recent years, there has been growing interest in the use of carbon admixtures to modify PMMA bone cements to improve their mechanical and functional properties. Nanomaterials such as carbon nanotubes or graphene oxide have received particular attention, but their practical application is limited by homogenisation difficulties and biosafety issues. Glassy carbon is an interesting alternative due to its high biocompatibility, chemical resistance, and thermal stability, but its effect on the properties of PMMA cements has not yet been comprehensively evaluated. The aim of this study was therefore to determine to what extent the size and concentration of glassy carbon particles affect the basic mechanical parameters of the composite. The obtained experimental results for compressive strength were highly consistent, with the coefficient of variation not exceeding 10% (Table 1). Only the Refobacin Plus series that exhibited complete degradation (*) of the cement due to GC admixing reached values with up to 25% scatter. In that cases, weakened compressive strength was approximately 20 MPa (with GC 0.4–12 µm at 5% *w*/*w*) and below 6 MPa (with GC 20–50 µm at ratios > 1% *w*/*w*). Such results, observed within individual series, confirm that the tests were carried out correctly.

**Figure 4:**
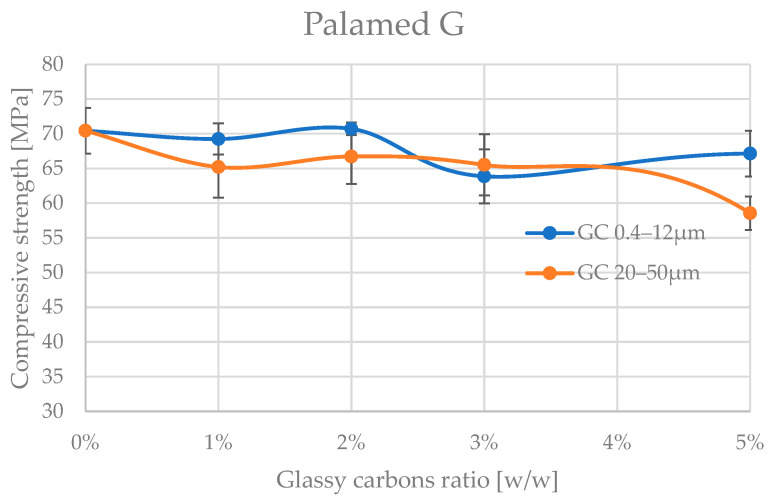
Compressive strength of Palamed bone cement admixture by glassy carbon.

**Figure 5:**
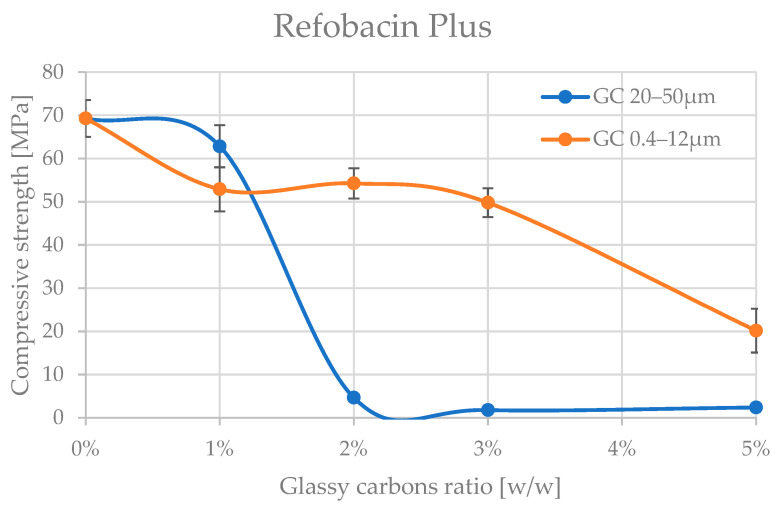
Compressive strength of Refobacin Plus bone cement admixture by glassy carbon.

The analyses of the obtained trends in compressive strength variation with increasing amount of admixture material are shown in Figure 4 and Figure 5, and the corresponding values are presented in Table 2 and Table 3.

A preliminary analysis of the results already shows that the glassy carbon granulation influences the degradation of the compressive strength of the two cements tested. The cements appear to be significantly more resistant to admixture with finer granulation of (0.4–12 μm). The differences in mechanical behaviour between Palamed^®^ and Refobacin Plus G cements may be due not only to the presence of GC alone but also to the different base formulation of these cements, which is why comparing the behaviour between the two materials was not the aim of this work. Refobacin contains the addition of an antibiotic (gentamicin), which can interact with glassy carbon to alter the curing properties and microstructure of the matrix. The presence of bioactive components may also affect the local pH and the degree of residual monomer release, resulting in a decrease in mechanical integrity [53,54,55].

Palamed G, even at 5% of this GC in the formulation, did not appear to be impaired. Refobacin Plus, on the other hand, recorded a deterioration in strength characteristics from as little as 1% of the GC in the formulation, with an average of up to approx. 25%. At 5% GC 0.4–12 μm, the weakening already reached up to 70% on average. Glassy carbon with a coarser grain size (20–50 μm) caused a much more pronounced reduction in compressive strength of the cements. Palamed G, for example, started to weaken noticeably only from approximately 4–5% admixture, reaching values about 17% lower at 5%. Refobacin Plus showed this behaviour much earlier. Above 1% admixture, the strength started to decrease drastically, and values below 10 MPa were reached from about 2% GC content in the composition. The observed significant deterioration of mechanical properties at larger GC fractions may be due to several overlapping physiochemical phenomena. Larger particles may disrupt the crosslinking of the polymer, leading to the formation of micropores and also acting as stress concentration sites, promoting crack initiation. In addition, large grains can absorb the heat of the exothermic reaction more efficiently, leading to uneven hardening of the cement at the microscale [56,57,58].

**Figure 6:**
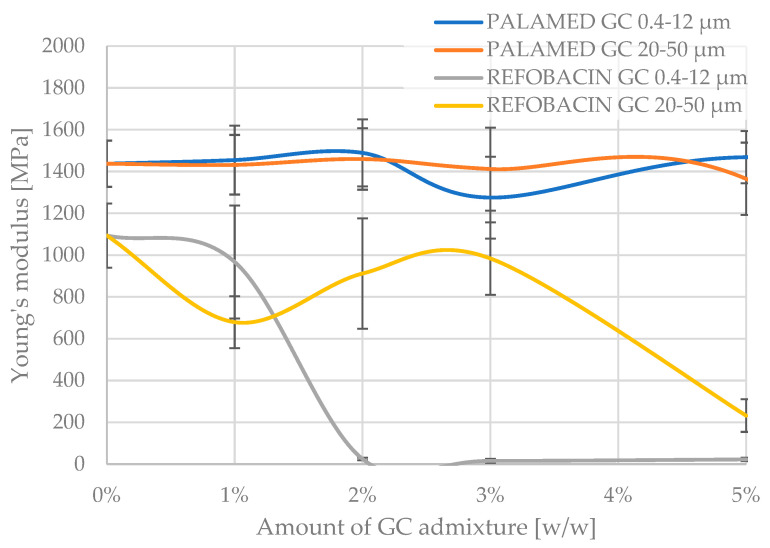
Young’s modulus of tested glassy carbon-loaded bone cements.

The Young’s modulus, which determines the longitudinal elasticity of the material, i.e., the resistance of the material to elastic deformation under compressive stresses, followed a similar pattern to the compressive strength, with the values recorded in the experiment showing greater scatter, as can be seen in Figure 6 below. Although the Young’s modulus values showed a larger scatter than the compressive strength, their analysis makes it possible to conclude on the effect of the GC additive on the stiffness of the composite. Particularly significant is the fact that for Palamed cement, despite the relative stability in strength, an increase in GC concentration of 20–50 µm resulted in a decrease in the stiffness of the material. This may indicate the formation of discontinuities in the matrix structure or the weakening of interfacial bonds. Palamed is a slightly stiffer cement and deformed less under compression. On average, the unmodified cement had approximately 30% higher modulus values. The obtained results, without further statistical analysis, do not show a change in modulus with increasing GC admixing in the two granulations tested, up to 5% GC in the cement composition. This partially corresponds with the results for compressive strength. Refobacin, as before, becomes more flexible quite quickly when admixed with 20–50 µm coarser carbon. The finer carbon clearly softens the cement only after approx. 3% in the composition.

The statistical analysis conducted ultimately confirms a degree of stability in the strength characteristics as the content of both types of glassy carbon increases in the Palamed cement formulation (Table 4). However, from a clinical perspective, loss of strength below the 20 MPa threshold, as occurred in some Refobacin series marked with “*” in Table 5, completely disqualifies these formulations for load-bearing applications. It is therefore of utmost importance to continue the search for optimal concentrations and GC fractions that will maintain the desired strength parameters without the risk of mechanical degradation. These results also indicate the need to implement fatigue and long-term stability tests for promising configurations. The observed reduction in mechanical performance—particularly for Refobacin Plus G with coarse GC particles—may be attributed to microstructural phenomena such as particle agglomeration and weak matrix adhesion. Larger GC particles likely acted as inert inclusions, disrupting polymer continuity and forming microvoids that reduce load transfer efficiency [59]. Their poor interfacial bonding with PMMA could have promoted early crack initiation. Additionally, localized thermal stress from uneven heat dissipation during curing may have further impaired structural integrity. These mechanisms explain the strength degradation trends observed. Although direct microstructural analyses (e.g., SEM/EDS) were not included, they are planned in future work to validate these hypotheses.

The results of the Palamed cement tests are not conclusive. Groups of homogeneous results, in the case of thinner carbon powder (0.4–12 μm) seem to cross over without definitively separating up to a 5% admixture proportion in the cement. As expected, coarser carbon at 5% in the composition leads to a disqualifying weakening of the cement.

Another important aspect that may influence the mechanical integrity and phase compatibility of bone cements is the surface chemistry of the filler particles. In PMMA-based composites, surface functionalization of inorganic particles via various methods has been widely applied to enhance interfacial adhesion with the polymer matrix [60]. For example, the silanization process involves chemical bonding between silane coupling agents and hydroxyl groups on the filler surface, forming Si–O–Si bridges that facilitate stronger interaction with the organic matrix. Studies have shown that silanization improves dispersion and significantly enhances the mechanical performance of PMMA composites. For example, silanized SiO_2_ nanoparticles increased the elongation at break by 136% compared to neat PMMA and improved interfacial adhesion reduced phase separation phenomena [61,62].

However, the effect of silanization is not unambiguously positive. At higher pH values, silanol groups on the silica surface tend to deprotonate, resulting in strong negative surface charges that may adversely affect filler dispersion and interfacial bonding [63,64]. Furthermore, even silanized particles such as hydroxyapatite (HAP) can exhibit poor compatibility with PMMA, as evidenced by persistent microstructural discontinuities and suboptimal bonding zones [65]. Thermal analyses have also indicated that the presence of silanized fillers may improve thermal stability, delaying the onset of degradation compared to non-functionalized composites. To validate the hypothesis regarding dispersion and interfacial bonding, scanning electron microscopy (SEM) and energy-dispersive X-ray spectroscopy (EDS) will be employed in follow-up studies to assess filler distribution, interfacial zone continuity, and particle–matrix compatibility.

Although silanization was not applied in the present study, the discussed findings suggest that future work should explore the potential of surface-modified GC particles to improve matrix compatibility and reduce mechanical degradation in PMMA cements.

A comparative analysis of the obtained Young’s modulus values for Palamed showed a lack of variability up to 5% of admixture, regardless of the type (thickness) of carbon admixed. Refobacin, on the other hand, significantly lost its stiffness above 2%, for carbon (0.4–12 μm) and at 5% for carbon (20–50 μm), according to the graphs.

Additional analyses carried out to determine the significance of the change in the characteristics of the cements tested in relation to admixing with glassy carbon of different granulations are shown in Table 6, showing statistically significant differences (red areas) between the samples.

The results presented so far mainly focus on mechanical properties; however, it may be equally important to investigate the interface between the GC particles and the PMMA matrix. In future work, it would be worthwhile to complement the analysis with SEM/EDS observations and DSC or TGA analysis to assess the effect of the additive on the exothermic reaction and the degree of monomer conversion. This will allow a more detailed explanation of the observed attenuation and potential influence of GC on the polymerisation process.

In the context of designing functional biomaterials, the ability to integrate the material into the host tissue (bio-integration) becomes an important criterion in addition to mechanical strength and chemical stability. Conventional PMMA cements as biologically inert materials do not directly promote osteointegration processes and do not form chemical bonds with bone. For this reason, various solid additives, including carbon admixtures, are being investigated that could support bone cell adhesion and the formation of a biological interface. Glassy carbon, due to its chemical inertness, microporous structure, and stability in a physiological environment, could be a favourable phase in the context of creating more bioactive cement surfaces. Although GC itself does not exhibit osteo-inductive activity, its structure may promote cell adhesion, especially in the presence of synergistic bioactive admixtures (e.g., HA and TCP). Addressing these aspects in future studies, including the evaluation of the expression of osteoblastic markers and the ability to form bone junctions in vitro and in vivo, may broaden the application of PMMA-GC cements as next-generation functional biomaterials.

The observed deterioration in mechanical performance, particularly in Refobacin Plus G cement with coarser GC particles, may be attributed to complex physicochemical interactions occurring at the cement–additive interface. One of the most plausible mechanisms involves the disruption of polymer chain crosslinking caused by the physical presence of larger GC particles, which can act as inert inclusions, reducingÍ matrix cohesion and promoting the initiation of microdefects under load. This is supported by literature findings indicating that oversized or poorly dispersed additives create interfacial voids and reduce the load transfer efficiency between phases [56,57,58]. In contrast, the finer GC particles (0.4–1.2 μm) appear to exhibit better compatibility with the PMMA matrix, maintaining compressive strength at low-to-moderate concentrations. This effect may stem from more uniform dispersion and a higher interfacial area per unit volume, which improves mechanical interlocking and stress distribution [66,67]. Moreover, the thermal conductivity of glassy carbon could partially alleviate localized overheating during polymerization, especially in denser PMMA matrices such as Palamed^®^, reducing internal stress build-up [68]. Although the thermal behaviour of PMMA cements is considered a critical aspect of clinical performance, especially due to the risk of thermal necrosis during polymerization, the present study did not include direct measurement of the polymerization exotherm. The hypothesis regarding the potential of glassy carbon (GC) to mitigate exothermic effects is based on its known high thermal conductivity and previous literature indicating that carbon-based additives, particularly with high specific surface area, may assist in heat dissipation during the curing process. However, without direct calorimetric or thermal imaging data, this aspect remains speculative in the context of our results. Future work will include differential scanning calorimetry (DSC) to quantify the exothermic behaviour during polymerization as well as thermogravimetric analysis (TGA) to assess the thermal stability and degradation patterns of the modified matrix. These methods will allow for validation of the hypothesized thermal effects of GC additives.

Importantly, in Refobacin Plus G, the presence of gentamicin may influence the curing kinetics and chemical microenvironment. Antibiotic particles can interfere with polymerization and potentially alter the interface between the organic matrix and GC particles, as previously reported [53,54,55]. This may also explain the rapid decline in strength at even low GC concentrations. Further microstructural investigations such as SEM/EDS and polymer conversion analysis via DSC are required to confirm these hypotheses and provide deeper insights into GC–PMMA interactions.

These findings underscore the dual role of GC not only as a structural modifier but also as a potential thermal and chemical regulator in the composite system. Understanding these mechanisms will be key to optimizing the formulation of future biofunctional PMMA bone cements [69].

From a clinical perspective, PMMA bone cements used in orthopaedic applications are expected to meet the ISO 5833:2002 standard, which requires a minimum compressive strength of 70 MPa. While unmodified Palamed^®^ and Refobacin Plus G cements comply with this criterion, the addition of coarse GC particles may reduce mechanical performance below this threshold, potentially disqualifying them from load-bearing use.

Various strategies have been proposed to improve PMMA performance, including fibre reinforcement, ceramic additives, and carbon nanostructures. Clinically, PMMA is widely used in arthroplasty and vertebral augmentation but remains biologically inert. As such, there is growing interest in materials like glassy carbon, which may not only support mechanical reinforcement but also reduce thermal necrosis and provide a structurally favourable interface for bone integration when combined with bioactive phases.

## 4. Conclusions

The study showed that the addition of glassy carbon (GC) particles to PMMA-based bone cements significantly affects their compressive strength and modulus of elasticity, with the nature and magnitude of this effect being highly dependent on grain size and admixture concentration. Particularly unfavourable effects were observed for coarser particles (20–50 μm), which led to a marked degradation of mechanical properties, especially in the Refobacin Plus G cement already at concentrations of the order of 1–2 wt.%. In contrast, finer particles (0.4–1.2 μm) at moderate concentrations (up to 3%) did not significantly adversely affect the mechanical properties of Palamed^®^ cement, suggesting their greater compatibility with the PMMA matrix.

The results emphasise the importance of precise selection of admixture parameters—both in terms of particle morphology and the characteristics of the base cement itself. Although glassy carbon has high biocompatibility, chemical resistance, and good mechanical performance, its use as an additive in acrylic cements requires careful matching to avoid weakening the material structure. Notably, even small differences in the composition of the cement, such as the presence of an antibiotic or a change in viscosity, can significantly alter the response of the composite to admixing.

From a biomaterials engineering perspective, the results obtained indicate that an appropriately selected glassy carbon fraction and concentration can support the creation of functional composites that combine mechanical requirements with potential biological benefits in an implant environment.

## 5. Limitations and Future Plans

Despite the significant results obtained, this study has some limitations that must be taken into account when interpreting the data. The main limitation of this study is the focus solely on compressive strength and Young’s modulus, without evaluating other clinically relevant mechanical parameters, such as bending strength, fracture toughness, creep resistance, or fatigue life. These parameters are crucial to understanding the long-term performance of bone cements under real physiological loads. Future experimental work will include three-point bending and fatigue tests under cyclic loading conditions to assess durability and crack propagation resistance. Increasing the sample size beyond the current group count (typically 8–10 per series) will be considered to enhance the robustness of statistical analysis and reduce the influence of experimental variability. Meanwhile, it is the complex and cyclic loads accompanying the daily use of endoprostheses under biological conditions that represent the primary challenge to the durability of bone cements.

It is important to acknowledge that the conclusions of this study are based on a limited set of experimental variants—specifically, two types of PMMA bone cements, two size ranges of GC particles, and five concentration levels. While these configurations provide an initial understanding of the mechanical effects of glassy carbon as an additive, they do not cover the full range of clinically relevant formulations or potential particle morphologies. Therefore, the generalization of the presented conclusions should be approached with caution. In future studies, the experimental matrix should be expanded to include a broader range of GC particle sizes, morphologies (e.g., flakes, spherical, porous, etc.), and a finer gradient of concentrations for a more comprehensive evaluation enabling more rigorous statistical modelling and a more definitive assessment of the suitability of glassy carbon for use in functional bone cement composites. Moreover, mechanical testing will be extended to include fracture toughness, creep, and fatigue analyses under simulated physiological conditions, allowing for a more complete mechanical characterization of GC-modified cements. In addition, future studies should include analyses of fatigue resistance, biological performance (e.g., osteoblast activity), and long-term degradation, validating the functional potential of the selected formulations under clinically relevant conditions.

An important limitation is also the lack of microstructural analysis of the samples (e.g., SEM/EDS) to verify the distribution of glassy carbon particles in the matrix and to identify possible defects such as micropores or zones of local stress concentration. Another important limitation is the lack of direct thermal characterization of the polymerization process. Although thermal effects were discussed in the context of the literature, no data on polymerization exotherm or thermal peak values were collected. This prevents a conclusive evaluation of the hypothesis that glassy carbon may mitigate excessive heat generation. Future work should include quantitative thermal analysis such as DSC, TGA, or thermal imaging during setting to assess the extent to which GC particles influence heat generation and thermal gradients in PMMA cements.

Therefore, further research should be directed towards the following:

- Evaluation of the fatigue properties and creep resistance of selected PMMA + GC composite formulations;
- Microstructure analysis using imaging methods (SEM and micro-CT);
- Thermal properties (DSC and TGA) and residual monomer release tests;
- Biocompatibility tests and evaluation of cytotoxicity and osteoblast activity in vitro;
- Simulations of physiological loads using numerical models (e.g., finite element method (FEM) or machine learning models) [70,71,72];
- Optimisation of the admixture mixing and homogenisation process under operating theatre-like conditions.

Additionally, the present study did not implement surface modification techniques such as silanization, which may enhance interfacial bonding and reduce phase separation in PMMA composites. Future research should include comparative studies with silanized GC or hybrid fillers to evaluate this effect.

Extending the scope of research to include the above aspects will allow a comprehensive assessment of the suitability of glassy carbon as an additive to bone cements and the determination of the limiting parameters of its use from the perspective of safety and clinical efficacy.

Further investigations are currently being conducted, involving the use of SEM/EDS to assess micro-structural interactions, DSC/TGA analyses for thermal profiling, and extended mechanical testing (bending, fracture, and fatigue). It is expected that these complementary studies will be submitted in a follow-up publication focused on microstructural–functional relationships in PMMA-GC composites. At this particular phase of the project, the incorporation of additional experimental data is not a viable option due to limitations in terms of resources and time. However, the proposed thermal, structural, and extended mechanical investigations are already in progress and will be presented in a separate, subsequent manuscript. These efforts are designed to systematically address the limitations identified in the present study.

## Figures and Tables

**Table 1:** Coefficient of variation of analysed series compressive strength (*—complete degradation).

Cement	GC Ratio GC Type	0%	1%	2%	3%	5%
Palamed G	GC 0.4–12 µm	4.7%	3.2%	1.3%	6.1%	4.9%
	GC 20–50 µm		6.8%	5.9%	6.8%	4.1%
Refobacin Plus	GC 0.4–12 µm	6.1%	9.6%	6.5%	6.7%	* 25.2%
	GC 20–50 µm		7.8%	* 17.5%	* 14.3%	* 9.9%

**Table 2:** Compressive strength values with standard deviation of Palamed bone cement.

GC Ratio GC Type	0%	1%	2%	3%	5%
GC 0.4–12 µm	70.4 ± 3.3 MPa	69.3 ± 2.2 MPa	70.7 ± 0.9 MPa	63.9 ± 3.9 MPa	67.2 ± 3.3 MPa
GC 20–50 µm	70.4 ± 3.3 MPa	65.2 ± 4.4 MPa	66.7 ± 3.9 MPa	65.5 ± 4.4 MPa	58.6 ± 2.4 MPa

**Table 3:** Compressive strength values with standard deviation of Refobacin Plus bone cement.

GC Ratio GC Type	0%	1%	2%	3%	5%
GC 0.4–12 µm	69.3 ± 4.3 MPa	52.9 ± 5.1 MPa	54.3 ± 3.5 MPa	49.8 ± 3.3 MPa	20.2 ± 5.1 MPa
GC 20–50 µm	69.3 ± 4.3 MPa	62.8 ± 4.9 MPa	4.7 ± 0.8 MPa	1.8 ± 0.3 MPa	2.4 ± 0.2 MPa

**Table 4:** Groups of statistically non-different results of compressive strength between Palamed cement admixed with different amounts of GC of different granularity.

	Palamed + GC 0.4–12 μm			Palamed + GC 20–50 μm			
GC Ratio	Compressive Strength [MPa]	1	2	Compressive Strength [MPa]	1	2	3
0%	70.45	X		70.45	X		
1%	68.52	X	X	65.22		X	
2%	70.71	X		67.83	X	X	
3%	65.19		X	66.45	X	X	
5%	67.56	X	X	58.55			X

**Table 5:** Groups of statistically non-different results of compressive strength between Refobacin cement admixed with different amounts of GC of different granularity (*—significantly degraded).

	Refobacin + GC 0.4–12 μm				Refobacin + GC 20–50 μm			
GC Ratio	Compressive Strength [MPa]	1	2	3	Compressive Strength [MPa]	1	2	3
0%	69.30	X			69.30	X		
1%	62.84		X		52.90		X	
2%	4.68 *			X	54.26		X	
3%	1.78 *			X	49.81		X	
5%	2.40 *			X	20.18 *			X

**Table 6:** The *p*-values (HSD test): differences between glassy carbon particle sizes: 20–50 µm vs. 0.4–1.2 µm (red: statistically significant differences, *p* < 0.05; blue: not significant).

Mechanical Parameter	Bone Cement	Amount of GC Admixture [*w*/*w*]			
		1%	2%	3%	5%
Compressive Strength	Palamed^®^ (Heraeus)	0.118	0.036	0.508	0.000
	Refobacin Plus G	0.014	0.000	0.000	0.000
Young’s modulus	Palamed^®^ (Heraeus)	0.801	0.748	0.258	0.267
	Refobacin Plus G	0.062	0.000	0.000	0.001

## Data Availability

The original contributions presented in the study are included in the article, further inquiries can be directed to the corresponding author.

## Abbreviations

The following abbreviations are used in this manuscript:

CNTs	Carbon nanotubes
DSC	Differential scanning calorimetry
EDS	Energy-dispersive X-ray spectroscopy
FEM	Finite element method
GO	Graphene oxide
HA	Hydroxyapatite
HSD test	Honestly significant difference test
ISO	International Organization for Standardization
MPa	Megapascal
MRT	Multiple range test
PMMA	Poly(methyl methacrylate)
SEM	Scanning electron microscopy
TCP	Tricalcium phosphate
TGA	Thermogravimetric analysis

## References

[1] Mounika C., Tadge T., Keerthana M., Velyutham R., Kapusetti G. (2023). Advancements in poly(methyl Methacrylate) bone cement for enhanced osteoconductivity and mechanical properties in vertebroplasty: A comprehensive review. Med. Eng. Phys..

[2] Quan Q., Gongping X., Ruisi N., Shiwen L. (2023). New Research Progress of Modified Bone Cement Applied to Vertebroplasty. World Neurosurg..

[3] Karpiński R., Szabelski J., Maksymiuk J. (2019). Seasoning Polymethyl Methacrylate (PMMA) Bone Cements with Incorrect Mix Ratio. Materials.

[4] Choryłek P. (2019). Vertebroplasty and kyphoplasty-advantages and disadvantages used bone cement of PMMA. J. Achiev. Mater. Manuf. Eng..

[5] Pandey J., Rajput S. (2025). Orthopedic Implants: Necessity of Coating, Their Advantages, and Challenges. J. Bio-Tribo-Corros..

[6] Ardelean A.I., Mârza S.M., Marica R., Dragomir M.F., Rusu-Moldovan A.O., Moldovan M., Pașca P.M., Oana L. (2024). Evaluation of Biocomposite Cements for Bone Defect Repair in Rat Models. Life.

[7] Karpinski R., Szabelski J., Maksymiuk J. (2018). Analysis of the properties of bone cement with respect to its manufacturing and typical service lifetime conditions. MATEC Web Conf..

[8] Lee C. (2005). The Mechanical Properties of PMMA Bone Cement. The Well-Cemented Total Hip Arthroplasty.

[9] Dunne N.J., Orr J.F., Mushipe M.T., Eveleigh R.J. (2003). The relationship between porosity and fatigue characteristics of bone cements. Biomaterials.

[10] Wang J.-S., Dunne N. (2008). Bone cement fixation: Acrylic cements. Joint Replacement Technology.

[11] Tzagiollari A., McCarthy H.O., Levingstone T.J., Dunne N.J. (2022). Biodegradable and Biocompatible Adhesives for the Effective Stabilisation, Repair and Regeneration of Bone. Bioengineering.

[12] Soleymani Eil Bakhtiari S., Bakhsheshi-Rad H.R., Karbasi S., Tavakoli M., Razzaghi M., Ismail A.F., RamaKrishna S., Berto F. (2020). Polymethyl Methacrylate-Based Bone Cements Containing Carbon Nanotubes and Graphene Oxide: An Overview of Physical, Mechanical, and Biological Properties. Polymers.

[13] Moldovan F. (2024). Role of Serum Biomarkers in Differentiating Periprosthetic Joint Infections from Aseptic Failures after Total Hip Arthroplasties. J. Clin. Med..

[14] Lin H., Gao Z., Shan T., Asilebieke A., Guo R., Kan Y., Li C., Xu Y., Chu J. (2024). A review on the promising antibacterial agents in bone cement–From past to current insights. J. Orthop. Surg. Res..

[15] Angelini J., Giuliano S., Russiani F., Lo Re F., Flammini S., Cadeo B., Martini L., Tascini C., Baraldo M. (2025). PK/PD Analysis of High-Dose Daptomycin Use in the Treatment of Bone and Joint Infections: Data from a Real-World Setting. Microorganisms.

[16] Robo C., Wenner D., Ubhayasekera S.J.K.A., Hilborn J., Öhman-Mägi C., Persson C. (2021). Functional Properties of Low-Modulus PMMA Bone Cements Containing Linoleic Acid. J. Funct. Biomater..

[17] Enache A.-V., Toader C., Onciul R., Costin H.P., Glavan L.-A., Covache-Busuioc R.-A., Corlatescu A.-D., Ciurea A.V. (2025). Surgical Stabilization of the Spine: A Clinical Review of Spinal Fractures, Spondylolisthesis, and Instrumentation Methods. J. Clin. Med..

[18] Wilczyński M., Bieniek M., Krakowski P., Karpiński R. (2024). Cemented vs. Cementless Fixation in Primary Knee Replacement: A Narrative Review. Materials.

[19] Phakatkar A.H., Shirdar M.R., Qi M., Taheri M.M., Narayanan S., Foroozan T., Sharifi-Asl S., Huang Z., Agrawal M., Lu Y. (2020). Novel PMMA bone cement nanocomposites containing magnesium phosphate nanosheets and hydroxyapatite nanofibers. Mater. Sci. Eng. C.

[20] Karpiński R., Szabelski J., Krakowski P., Jonak J., Falkowicz K., Jojczuk M., Nogalski A., Przekora A. (2024). Effect of various admixtures on selected mechanical properties of medium viscosity bone cements: Part 2—Hydroxyapatite. Compos. Struct..

[21] Jalaludeen A.M., Ramakrishnan R., Gunasekaran S.S., Thajuddin N., Selvam M.K., Ali B.M.S., Dua R., Ramakrishnan P., Ramesh M.D., Vinayagam S. (2024). Advancements in hydroxyapatite synthesis and surface modifications for emerging biomedical applications. Inorg. Chem. Commun..

[22] Karpiński R., Szabelski J., Krakowski P., Jonak J., Falkowicz K., Jojczuk M., Nogalski A., Przekora A. (2024). Effect of various admixtures on selected mechanical properties of medium viscosity bone cements: Part 1—α/β tricalcium phosphate (TCP). Compos. Struct..

[23] Ghasemi F., Jahani A., Moradi A., Ebrahimzadeh M.H., Jirofti N. (2023). Different Modification Methods of Poly Methyl Methacrylate (PMMA) Bone Cement for Orthopedic Surgery Applications. Arch. Bone Jt. Surg..

[24] Wekwejt M., Chen S., Kaczmarek-Szczepańska B., Nadolska M., Łukowicz K., Pałubicka A., Michno A., Osyczka A.M., Michálek M., Zieliński A. (2021). Nanosilver-loaded PMMA bone cement doped with different bioactive glasses—Evaluation of cytocompatibility, antibacterial activity, and mechanical properties. Biomater. Sci..

[25] Mansoori-Kermani A., Mashayekhan S., Kermani F., Abdekhodaie M.J. (2023). The effect of tricalcium silicate incorporation on bioactivity, injectability, and mechanical properties of calcium sulfate/bioactive glass bone cement. Ceram. Int..

[26] Khandaker M., Riahinezhad S., Jamadagni H., Morris T., Coles A., Vaughan M. (2017). Use of Polycaprolactone Electrospun Nanofibers as a Coating for Poly(methyl methacrylate) Bone Cement. Nanomaterials.

[27] Cai P., Lu S., Yu J., Xiao L., Wang J., Liang H., Huang L., Han G., Bian M., Zhang S. (2023). Injectable nanofiber-reinforced bone cement with controlled biodegradability for minimally-invasive bone regeneration. Bioact. Mater..

[28] Shirdar M., Taheri M., Qi M.-L., Gohery S., Farajpour N., Narayanan S., Foroozan T., Sharifi-Asl S., Shahbazian-Yassar R., Shokuhfar T. (2021). Optimization of the Mechanical Properties and the Cytocompatibility for the PMMA Nanocomposites Reinforced with the Hydroxyapatite Nanofibers and the Magnesium Phosphate Nanosheets. Materials.

[29] Paz E., Ballesteros Y., Abenojar J., Del Real J.C., Dunne N.J. (2019). Graphene Oxide and Graphene Reinforced PMMA Bone Cements: Evaluation of Thermal Properties and Biocompatibility. Materials.

[30] Karpiński R., Szabelski J., Krakowski P., Jonak J., Falkowicz K., Jojczuk M., Nogalski A., Przekora A. (2024). Effect of various admixtures on selected mechanical properties of medium viscosity bone cements: Part 3—Glassy carbon. Compos. Struct..

[31] Ormsby R., McNally T., Mitchell C., Halley P., Martin D., Nicholson T., Dunne N. (2011). Effect of MWCNT addition on the thermal and rheological properties of polymethyl methacrylate bone cement. Carbon.

[32] Ormsby R., McNally T., Mitchell C., Dunne N. (2010). Incorporation of multiwalled carbon nanotubes to acrylic based bone cements: Effects on mechanical and thermal properties. J. Mech. Behav. Biomed. Mater..

[33] Gonçalves G., Cruz S.M., Grácio J., Marques P.A., Ramírez-Santillán C., Vallet-Regí M., Portolés M.-T. (2012). New bioactive PMMA-hydroxyapatite based bone cement reinforced with graphene oxide. Graphene.

[34] Ormsby R.W., Modreanu M., Mitchell C.A., Dunne N.J. (2014). Carboxyl functionalised MWCNT/polymethyl methacrylate bone cement for orthopaedic applications. J. Biomater. Appl..

[35] Ormsby R., McNally T., Mitchell C., Dunne N. (2010). Influence of multiwall carbon nanotube functionality and loading on mechanical properties of PMMA/MWCNT bone cements. J. Mater. Sci. Mater. Med..

[36] Wang C., Yu B., Fan Y., Ormsby R.W., McCarthy H.O., Dunne N., Li X. (2019). Incorporation of multi-walled carbon nanotubes to PMMA bone cement improves cytocompatibility and osseointegration. Mater. Sci. Eng. C.

[37] Gonçalves G., Marques P.A.A.P., Barros-Timmons A., Bdkin I., Singh M.K., Emami N., Grácio J. (2010). Graphene oxide modified with PMMA via ATRP as a reinforcement filler. J. Mater. Chem..

[38] Pahlevanzadeh F., Bakhsheshi-Rad H.R., Ismail A.F., Aziz M., Chen X.B. (2019). Development of PMMA-Mon-CNT bone cement with superior mechanical properties and favorable biological properties for use in bone-defect treatment. Mater. Lett..

[39] Uskoković V. (2021). A historical review of glassy carbon: Synthesis, structure, properties and applications. Carbon Trends.

[40] Wang Y., Wen X., Zhang P., Yu H., Wang L., Li J., Jiang W., Liu X. (2025). Bio-glassy carbon bulks via powder metallurgy enabled by phase evolution of nanodiamond. Ceram. Int..

[41] Hucke E.E., Fuys R.A., Craig R.G. (1973). Glassy carbon: A potential dental implant material. J. Biomed. Mater. Res..

[42] Choryłek P., Postawa P. (2020). The effect of glassy carbon and cancellous bone admixture on performance and thermal properties of acrylic bone cements. Arch. Mater. Sci. Eng..

[43] Choryłek P. (2020). Comparison of selected properties of cements modified with glassy carbon and cancellous bone. Arch. Mater. Sci. Eng..

[44] Glassy Carbon Powder, Type 1, 20–50 µm, Alfa AesarTM. https://www.thermofisher.com/order/catalog/product/041258.30?SID=srch-srp-041258.30.

[45] SPI Supplies (Structure Probe, Inc.) Sigradur K Brand Glassy (Vitreous) Carbon Powder Spherical. https://www.2spi.com/item/z4204gcps/.

[46] Kurzweil P. (2025). Supercapacitors|Carbon Technologies. Encyclopedia of Electrochemical Power Sources.

[47] Tanzi M.C., Farè S., Candiani G. (2019). Organization, Structure, and Properties of Materials. Foundations of Biomaterials Engineering.

[48] HTW Hochtemperatur-Werkstoffe GmbH (2023). Technical Data Sheet: Glassy Carbon Powders (GCmb Series).

[49] (2002). Implants for Surgery—Acrylic Resin Cements.

[50] Szabelski J., Karpiński R., Krakowski P., Jojczuk M., Jonak J., Nogalski A. (2022). Analysis of the Effect of Component Ratio Imbalances on Selected Mechanical Properties of Seasoned, Medium Viscosity Bone Cements. Materials.

[51] Szabelski J., Karpiński R., Krakowski P., Jonak J. (2021). The Impact of Contaminating Poly (Methyl Methacrylate) (PMMA) Bone Cements on Their Compressive Strength. Materials.

[52] Karpiński R., Szabelski J., Krakowski P., Jonak J. (2020). Effect of Physiological Saline Solution Contamination on Selected Mechanical Properties of Seasoned Acrylic Bone Cements of Medium and High Viscosity. Materials.

[53] Liawrungrueang W., Ungphaiboon S., Jitsurong A., Ingviya N., Tangtrakulwanich B., Yuenyongviwat V. (2021). In vitro elution characteristics of gentamicin-impregnated Polymethylmethacrylate: Premixed with a second powder vs. liquid Lyophilization. BMC Musculoskelet. Disord..

[54] Aydınoğlu A. (2024). Enhancing orthopedic outcomes: A comparative analysis of gentamicin sulphate and nanosilver in bone cement. Heliyon.

[55] Robu A., Antoniac A., Grosu E., Vasile E., Raiciu A.D., Iordache F., Antoniac V.I., Rau J.V., Yankova V.G., Ditu L.M. (2021). Additives Imparting Antimicrobial Properties to Acrylic Bone Cements. Materials.

[56] Lou C., Xu J., Wang T., Ren W. (2021). Microstructure and pore structure of polymer-cement composite joint sealants. Sci. Rep..

[57] Świeczko-Żurek B., Zieliński A., Bociąga D., Rosińska K., Gajowiec G. (2022). Influence of Different Nanometals Implemented in PMMA Bone Cement on Biological and Mechanical Properties. Nanomaterials.

[58] Khandaker M., Vaughan M., Morris T., White J., Meng Z. (2014). Effect of additive particles on mechanical, thermal, and cell functioning properties of poly(methyl methacrylate) cement. Int. J. Nanomed..

[59] Bain E.D., Mrozek R.A., Lenhart J.L. (2017). Role of weak particle-matrix interfacial adhesion in deformation and fracture mechanisms of rigid particulate-filled poly(methyl methacrylate). Mech. Mater..

[60] Meng Q., Kuan H.-C., Araby S., Kawashima N., Saber N., Wang C.H., Ma J. (2014). Effect of interface modification on PMMA/graphene nanocomposites. J. Mater. Sci..

[61] Kim G.H., Hwang S.W., Jung B.N., Kang D., Shim J.K., Seo K.H. (2020). Effect of PMMA/Silica Hybrid Particles on Interfacial Adhesion and Crystallization Properties of Poly(lactic acid)/Block Acrylic Elastomer Composites. Polymers.

[62] Shi S.-C., Zeng X.-X. (2024). Silica silanization graft-strengthening bone cement poly(methyl methacrylate): Process and dynamic mechanical properties. Mater. Res. Express.

[63] Shen Z., Wang T., Luo J., Liu R., Ngai T., Sun G. (2024). Unraveling the Complexities of Silica Nanoparticle Adsorption onto Polymer Latexes in Pickering Emulsion Polymerization. Langmuir.

[64] Colver P.J., Colard C.A.L., Bon S.A.F. (2008). Multilayered Nanocomposite Polymer Colloids Using Emulsion Polymerization Stabilized by Solid Particles. J. Am. Chem. Soc..

[65] Tham D.Q., Huynh M.D., Linh N.T.D., Van D.T.C., Cong D.V., Dung N.T.K., Trang N.T.T., Lam P.V., Hoang T., Lam T.D. (2021). PMMA Bone Cements Modified with Silane-Treated and PMMA-Grafted Hydroxyapatite Nanocrystals: Preparation and Characterization. Polymers.

[66] Kausch H.H., Michler G.H. (2007). Effect of nanoparticle size and size-distribution on mechanical behavior of filled amorphous thermoplastic polymers. J. Appl. Polym. Sci..

[67] Miola M., Lucchetta G., Verné E. (2023). Physical, Mechanical, and Biological Properties of PMMA-Based Composite Bone Cement Containing Silver-Doped Bioactive and Antibacterial Glass Particles with Different Particles Sizes. Materials.

[68] Ferrer-Argemi L., Cisquella-Serra A., Madou M., Lee J. Temperature-Dependent Electrical and Thermal Conductivity of Glassy Carbon Wires. Proceedings of the 2018 17th IEEE Intersociety Conference on Thermal and Thermomechanical Phenomena in Electronic Systems (ITherm).

[69] Vieira L.D.S. (2022). A review on the use of glassy carbon in advanced technological applications. Carbon.

[70] Fada R., Shahgholi M., Azimi R., Babadi N.F. (2024). Estimation of Porosity Effect on Mechanical Properties in Calcium Phosphate Cement Reinforced by Strontium Nitrate Nanoparticles: Fabrication and FEM Analysis. Arab. J. Sci. Eng..

[71] Machrowska A., Szabelski J., Karpiński R., Krakowski P., Jonak J., Jonak K. (2020). Use of Deep Learning Networks and Statistical Modeling to Predict Changes in Mechanical Parameters of Contaminated Bone Cements. Materials.

[72] Machrowska A., Karpiński R., Jonak J., Szabelski J., Krakowski P. (2020). Numerical prediction of the component-ratio-dependent compressive strength of bone cement. Appl. Comput. Sci..

